# Genetic and epigenetic variations contributed by Alu retrotransposition

**DOI:** 10.1186/1471-2164-12-617

**Published:** 2011-12-20

**Authors:** Alexandre de Andrade, Min Wang, Maria F Bonaldo, Hehuang Xie, Marcelo B Soares

**Affiliations:** 1 Falk Brain Tumor Center, Cancer Biology and Epigenomics Program, Children's Memorial Research Center, Chicago IL 60614-3394, USA; 2 Department of Pediatrics; Feinberg School of Medicine, Northwestern University, Chicago IL 60614-3394, USA

## Abstract

**Background:**

*De novo *retrotransposition of Alu elements has been recognized as a major driver for insertion polymorphisms in human populations. In this study, we exploited Alu-anchored bisulfite PCR libraries to identify evolutionarily recent Alu element insertions, and to investigate their genetic and epigenetic variation.

**Results:**

A total of 327 putatively recent Alu insertions were identified, altogether represented by 1,762 sequence reads. Nearly all such *de novo *retrotransposition events (316/327) were novel. Forty-seven out of forty-nine randomly selected events, corresponding to nineteen genomic loci, were sequence-verified. Alu element insertions remained hemizygous in one or more individuals in sixteen of the nineteen genomic loci. The Alu elements were found to be enriched for young Alu families with characteristic sequence features, such as the presence of a longer poly(A) tail. In addition, we documented the occurrence of a duplication of the AT-rich target site in their immediate flanking sequences, a hallmark of retrotransposition. Furthermore, we found the sequence motif (TT/AAAA) that is recognized by the ORF2P protein encoded by LINE-1 in their 5'-flanking regions, consistent with the fact that Alu retrotransposition is facilitated by LINE-1 elements. While most of these Alu elements were heavily methylated, we identified an Alu localized 1.5 kb downstream of TOMM5 that exhibited a completely unmethylated left arm. Interestingly, we observed differential methylation of its immediate 5' and 3' flanking CpG dinucleotides, in concordance with the unmethylated and methylated statuses of its internal 5' and 3' sequences, respectively. Importantly, TOMM5's CpG island and the 3 Alu repeats and 1 MIR element localized upstream of this newly inserted Alu were also found to be unmethylated. Methylation analyses of two additional genomic loci revealed no methylation differences in CpG dinucleotides flanking the Alu insertion sites in the two homologous chromosomes, irrespective of the presence or absence of the insertion.

**Conclusions:**

We anticipate that the combination of methodologies utilized in this study, which included repeat-anchored bisulfite PCR sequencing and the computational analysis pipeline herein reported, will prove invaluable for the generation of genetic and epigenetic variation maps.

## Background

Repetitive elements constitute over 50% of the human genomic sequence [1]. The most prevalent repeats are the Alu family of SINEs, which comprise approximately 10% of the human genome. A typical Alu element is approximately 300 bp long and contains two almost identical arms separated by an A-rich sequence. The ancestor of the Alu monomer is the 7 SL RNA gene, which encodes the RNA component of the signal recognition particle (SRP) that is involved in the translocation of newly synthesized proteins [2,3]. Similar to the 7 SL gene, Alu elements with intact promoters - namely A and B boxes - may be transcribed by RNA polymerase III [2,4]. With the aid of the LINE-encoded retrotransposition machinery, Alu transcripts gain mobility and expand in genomes through a process involving reverse transcription and integration [5].

Alu retrotransposition has been an important molecular evolutionary force reshaping the primate genomes [6]. The expansion of the Alu elements in the primate genomes is dated at least 60 million years ago [7]. Based on their evolutionary history, Alu elements can be classified in three major subfamilies: AluJ, AluS, and AluY [8]. Among them, the youngest Alu elements - AluY and its variants AluYa-g - remain very active, and exhibit the highest rate of retrotransposition in the human genome [9-12]. While several recent studies have shown that LINE-1 elements contribute substantially to the structural variations observed in the human genome [13-15], the retrotransposition rate of Alu elements is ten times higher than that of LINE-1, with an estimated new insertion at every 21 births [16].

Decades of research have demonstrated that Alu elements play important roles in the genome and transcriptome [17-20]. Alu elements may contribute a large number of transcription factor binding sites [21], some of which may serve as enhancers involved in tissue development [22,23]. In addition, some Alu elements may be expressed and Alu transcription affects nearby gene expression, distal gene expression, and global translation. For instance, the expression of an Alu in the promoter of an epsilon-globin gene was found to negatively regulate globin gene expression by transcriptional interference [24]. Recently, Alu RNA was found to be a modular transacting repressor of mRNA transcription [25]. Interestingly, such transcriptional suppression was found to be specific and limited to certain genes. Alu RNAs also affect translational initiation and were found to form stable, discrete complexes with the double-stranded RNA-activated kinase PKR, and to antagonize PKR activation [26]. Transcription derepression of otherwise active Alu elements, which so often reside within genes, may lead to the formation of double-strand RNA - if in antisense orientation - and ultimately to heterochromatinization and silencing of the gene [27].

One of the key mechanisms controlling Alu expression is DNA methylation. The human genome has approximately 28 million CpG dinucleotides, 7 million of which are found within Alu elements [1]. In most somatic tissues, the CpG dinucleotides within the Alu sequence are heavily methylated to suppress Alu expression [28,29]. It has been demonstrated that the A and B boxes (5-16 bp, and 75-84 bp from the 5' terminus, respectively) are critical cis-elements for Alu expression. In particular, methylation of the B box is thought to inhibit protein binding and hence block Alu transcription [30]. Albeit not sufficient, demethylation and consequently transcription of Alu elements is required for occurrence of *de novo *retrotransposition [28]. Methylated CpGs can undergo deamination and thereby lead to mutations that render them unable for retrotransposition [8,9].

Although much effort has been made to identify structural variations resulting from Alu integration, much less is known with regard to the epigenetic status of newly inserted elements and of their flanking genomic sequences. Here we report the utilization of an Alu-anchored bisulfite PCR strategy to generate methylation maps for thousands of Alu elements in human cerebellum and in ependymomas [31,32]. In this approach, most of the targeted Alu elements are members of the active AluY subfamilies. In this study, we analyzed the aforementioned datasets to identify newly integrated Alu elements, to investigate sequence characteristics and commonalities of their integration sites, to uncover their methylation statuses, and to determine whether the methylation patterns of the sequences surrounding their integration sites would be altered in the alleles harboring the insertion in individuals hemizygous for the Alu retrotransposon.

## Results

### Identification of recent Alu insertions

The method developed by Xie and colleagues was initially designed to generate a methylation map for a subset of young Alu elements [32]. The strategy applied a primer targeting CpG-rich Alu repeats to simultaneously amplify thousands of Alu elements and their 5' flanking sequences. Unequivocal mapping of these repeats was therefore achieved through their - most often unique - 5' flanking sequences. Eight Alu libraries were derived with this strategy, six from ependymomas and two from normal brain tissues [31,32].

**Figure 1:**
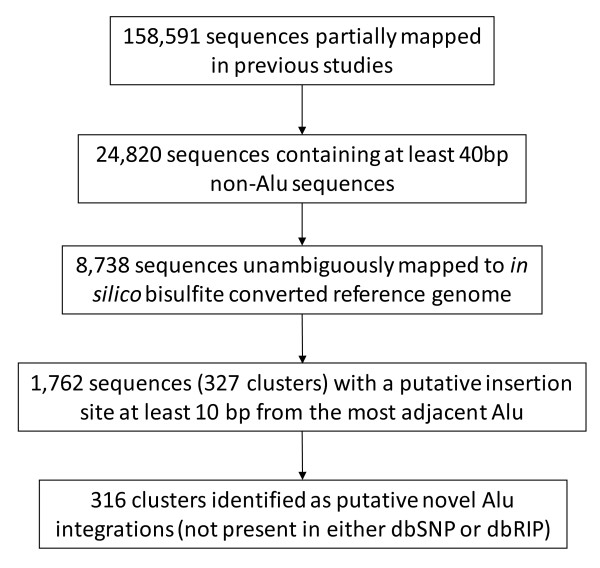
**Computational pipeline developed to identify putative Alu insertions from Alu anchored bisulfite PCR libraries**.

In previous studies, a number of sequence reads from these libraries could only be partially mapped to the human reference sequence. In order to determine whether any of these sequence reads corresponded to a novel Alu integration event, i.e. one that was not yet documented in the UCSC database, we designed a computational pipeline to reanalyze these datasets (Figure 1). For 158,591 sequence reads partially mapped in previous studies, we first masked Alu sequences and then selected the ones containing at least 40 bp of 5' flanking sequences. A total of 24,820 sequence reads were thus identified. The Alu flanking sequences were then extracted from these reads and subjected to Megablast against *in silico *bisulfite converted human reference genome sequence. Unambiguous mapping was achieved for 8,738 sequence reads. As expected, the majority of these reads (79.8%) mapped to genomic sequences adjacent to an Alu element. Further examination of the remaining 1,762 sequences reads (Additional File 1, Table S1) enabled their grouping into 327 clusters according to their genomic coordinates (Additional File 2, Table S2). It is noteworthy that due to the highly stringent mapping criteria applied in our previous studies [31,32], a few mismatches in the alignments between the reference genomic sequence and the sequences generated from the Alu libraries were sufficient to lead to their classification as "partially mapped" reads.

We examined the distribution of the 327 clusters comprised of 1,762 sequences reads in eight Alu-anchored bisulfite PCR libraries (Table 1). Out of 327 clusters, 163 clusters (49.8%) were found to be supported by more than one sequence read and 87 clusters (26.7%) were found to be present in more than one library. Among these 87 clusters, 56 clusters (64.4%) were found in both normal and tumor tissues. This indicates that a majority of these putative insertions are not associated with tumorigenesis and/or cancer progression. In addition, a library derived from a normal brain tissue contributed 159 clusters (48.6% of the total 327 clusters) with 692 sequence reads (39.3% of 1,762 total sequence reads), while a library derived from a relapsed aggressive ependymoma only contributed 16 clusters with 23 sequence reads. Based on the difference in number of sequence reads generated from each library, we normalized - for each library - the number of putative insertions that were identified, to the number of Alu repeats that were successfully mapped to the reference genome. No significant difference was observed in this ratio between normal and tumor tissues (p = 0.34, t-Test).

**Table 1:** 327 clusters comprised in 1,762 sequence reads.

Sample ID*	NC1	NC2	PA1	PA2	PA3	PA4	PA5	RL	Sum
sequenceRead_mapped	245,825	460,438	256,014	238,633	336,103	283,730	276,505	245,600	2,342.848

genomeRegion_mapped	31,871	47,071	31,683	33,760	41,485	33,937	33,052	36,957	289,816 (140,865**)

sequenceRead_Alu insertion	56	692	78	216	453	203	23	41	1,762

genomeRegion_Alu insertion	28	159	36	61	113	62	16	23	498 (327**)

Ratio (genomeRegion_AluInsertion/ genomeRegion_mapped)	0.09	0.34	0.11	0.18	0.27	0.18	0.05	0.06	

*NC1: normal cerebellum; and NC2: normal 4^th ^ventricle lining tissue; PA1, PA2, PA3, PA4, and PA5: primary ependymoma tumor; RL: ependymoma tumor relapsed from PA3.

**Number of non-redundant clusters.

To investigate whether these putatively new Alu insertions had been identified in previous studies, we extracted 1,763 and 795 known polymorphic Alu elements from dbSNP (The Single Nucleotide Polymorphism database, NCBI) [33] and dbRIP (Database of Retrotransposon Insertion Polymorphisms) [34], respectively. This analysis revealed that 316 of the 327 clusters were novel, i.e. they corresponded to yet undocumented *de novo *retrotransposition events. The putative integration sites of 140 of such clusters (42.8%) were found to localize to intronic regions, except for one, which mapped to the 3'-UTR of TOMM40, a gene that codes for the translocase of the mitochondrial outer membrane (TOM) complex. We further analyzed these genes with NCBI's DAVID functional annotation tool to examine whether any specific gene category was more likely to harbor these Alu insertions. One hundred thirty-two genes were found annotated in the NCBI database. Compared to all genes annotated in the human genome, no significant enrichment was identified for this set of 132 genes in terms of biological process, cellular localization or molecular function (Additional File 2, Table S2).

### Verification of recent Alu insertions

To validate the evolutionarily recent Alu *de novo *retrotransposition events identified in this study, we randomly selected twenty-one genomic loci encompassing such putative new Alu insertions. For each genomic locus, we designed primers based on the upstream and downstream sequences surrounding the predicted integration sites. With these primers, the PCR products were expected to be ~120 bp (without Alu insertion) or ~420 bp (with Alu insertion). Due to the diploidy of the human genome, three kinds of PCR results were expected: (1) hemizygous Alu insertion: PCR products of two different sizes were expected, one fragment with the Alu insertion and another without it (spanning ~420 bp and ~120 bp, respectively); (2) homozygous Alu insertion: only one PCR product was expected, this fragment containing an Alu element (spanning ~420 bp); (3) nulizygous Alu insertion: no Alu insertion was present in either homologous chromosome, hence just one small PCR product (spanning ~120 bp) was expected.

**Figure 2:**
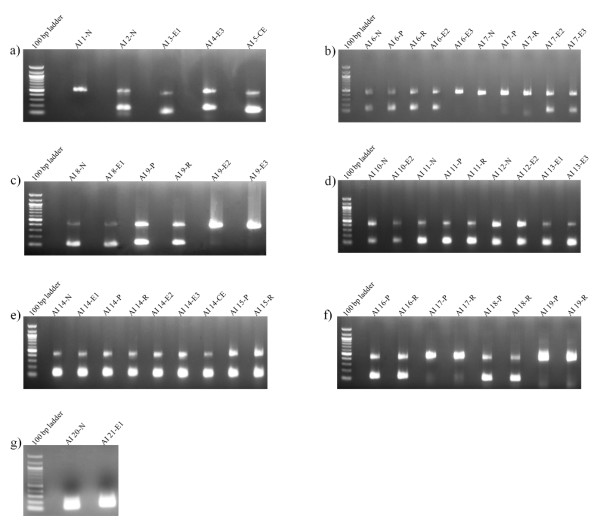
**PCR validation of putative Alu insertions (a-g)**. The Alu insertions were sorted based on genomic coordinates. The Alu insertions were named AI1 through AI21. N-normal brain tissue DNA; E1, E2, and E3-brain tumor tissue (ependymoma) DNA from different individuals; P and R- ependymoma DNA, P is primary and R is relapsed tumor from the same individual.

The Alu insertions were successfully verified for forty-seven out of forty-nine cases representing nineteen genomic loci (Figure 2). To ensure that the regions amplified by PCR were indeed new Alu insertions, for each locus, PCR products were cloned and sequence-verified. The sequences representing these nineteen genomic loci were submitted to GenBank. Their accession numbers are: [HQ709117, HQ709118, HQ709119, HQ709120, HQ709121, HQ709122, HQ709123, HQ709124, HQ709125, HQ709126, HQ709127, HQ709128, HQ709129, HQ709130, HQ709131, HQ709132, HQ709133, HQ709134, HQ709135]. Fourteen out of the nineteen insertion events were predicted to occur in more than one individual. Interestingly, we found that nine out of these fourteen insertions were hemizygous in all individuals examined - i.e., the Alu insertion only occurred in one of the two homologous chromosomes. The remaining five insertions were hemizygous for some individuals and homozygous for others. From a total of forty-seven Alu insertions, thirty-six were found to be hemizygous and eleven were found to be homozygous. The fact that the majority of the insertions have remained in hemizygosity in the genome may be interpreted as suggestive of their recent evolutionary origin. However, that will remain speculative until populational studies are performed.

### Genomic features and sequence characteristics of Alu elements and their flanking sequences

It has been shown that polymorphic Alu elements and their flanking sequences may share some distinct sequence features [34,35]. The Alu transcripts derived from the ones with conserved structure would interact productively with SRP9/15 host proteins and gain the ability to retrotranspose [12]. The AluY subfamily and its variants Yc1, Yc2, Ya5, Ya5a2, Ya8, Yd8, Yb8, and Yb9, are the ones considered to be very active due to the conservation of its structure. To conclude the analysis, we classified the Alu elements identified in this study according to its family of origin. We found that the new insertions identified in this study belong to the relatively recent family of AluY elements or to the subfamilies AluYa5, AluYb8, AluYb9, and AluYg6. It has also been shown that the occurrence of a longer poly(A) tail might facilitate Alu retrotransposition [35]. Our analysis revealed that all twenty-two new Alu insertions that were sequence verified in this study have an A-tail that ranged from 11 to 45 nucleotides, with an average length of 29 bp.

Alu retrotransposition is facilitated by LINE-1 elements. LINE elements encompass two open reading frames, namely ORF1 and ORF2P. ORF1 encodes a non-specific RNA binding protein, and ORF2P encodes an endonuclease and a reverse transcriptase. During the process of retrotransposition ORF2P cleaves genomic DNA at a degenerate consensus sequence (TT/AAAA). Accordingly, the presence of a TT/AAAA sequence motif in the 5'-flanking region seems essential for Alu insertion [5,36,37]. The Alu insertion site is generated by a single-strand break that occurs in the target DNA made by ORF2P. The mechanism of Alu insertion is called Target Primed Reverse Transcription (TPRT) [8,37]. Indeed, we were able to document the occurrence of this sequence motif - either a perfect match or a highly similar sequence - in the 5' flanking regions of all new Alu insertions that were sequence-verified in this study. For the nineteen Alu insertions identified in this study, the characteristic sequence features of Alu and flanking sequences are summarized in Table 2.

**Table 2:** Sequence features of newly inserted Alu repeats.

Sample	Chromosome coordinate	Alu subfamily	TSD	Putative cleavage site	Poly-A sequence
AI-1	chr15:61216453-61216621	AluY	AAGAAATGTTCT	TTAA	CTCAAAAAAAAAAAAAAAGAAAAAAAAAAAAGAAAAAAGAAAT

AI-2	chr5:139595078-139595241	AluYb8	TAAATTACAGA	TTAAA	CTCAAAAAAAAAAAAAAAATAAATAAATAAATAAATTA

AI-3	chr4:41598260-41598327	AluY	AAGTACATGTGG	TGGAA	CTCAAAAAAAAAAAAAAAAAAAAAAAAAAAAAGAAGT

AI-4	chr13:23662855-23663027	AluYa5	CATCTG	TTAAAA	CTCCGTCTCAAAAAAAAAAAAAACAAAAAAAAAAACAAAAAAAAAAAACATCT

AI-5	chr15:28179309-28179438	AluY	ATAAAACATGGTCTG	TATAAAA	CTCAAAAAAAAAAAAAAAATAAAAAAAAATAAATAAAAAAATAAAACAT

AI-6	chr12:32076361-32076491	AluYg6	GAAATAATTGATCT	TGAAA	CTCAAAAAAAAAAAAAAAAAAAAAGAAATAAT

AI-7	chr11:130675880-130675924	AluY	AAAAAGAAGC	TTAAAA	CTCAAAAAAAAAAAAAAAAAAAAGAAGCA

AI-8	chr5:141758572-141758694	AluYb8	AAAAATGGGGATT	TTAAAA	CTCAAAAAAAAAAAAAAAAAATGGGGA

AI-9	chr10:107891481-107891638	AluYg6	CGTGTGCTC	TTAAAA	CTCAAAAAAAAAAACGTGTG

AI-10	chr10:72605338-72605440	AluYg6	AAGAAGGTA	TAA	CTCAAAAAAAAAAAAAAAAAAAAAAAAAAAAAAAGAAGGT

AI-11	chr2:48276482-48276601	AluYb8	AGAAATTCAAATGCA	TTA	CTCAAAAAAAAAAAAAAAAAAAAAAAAAAAAAAAAAAAAAAAAAAAAGAAAT

AI-12	chr5:16716576-16716677	AluYg6	AAGAAGTATGACAG	TAA	CTCAAAAAAAAAAAAAAAAAAAAGAAGTAT

AI-13	chr12:24518543-24518646	AluY	AAAAAAGTATTAATCA	TTAAAA	CTCAAAAAAAAAAAAAAAAAAAAAAAAAAAAAAAAAAAAAAAAGTAT

AI-14	chr6:57403535-57403610	AluY	TCCTA	TAAT	CTCAAAAAAAAAAAAAAAAAAAAAAAAAAAAAAAAAAAAATTCC

AI-15	chr2:9888790-9888862	AluYb8	CACACCCGTG	TAA	CTCAAAAAAAAAAAAAAAAAAAAAAAAAAAAAAAACACAC

AI-16	chr9:1631754-1631884	AluYb8	AAGAA	CAAA	CTCAAAAAAAAAAAAAAAAAAAAAGAAAACA

AI-17	chr4:139225139-139225274	AluY	GAGTTTTTAAACATCT	TTAAA	CTCAAAAAAAAAAAAAAAAAAAAAAAAAAAAAAAAAAAAAAAAAAAAGGAGT

AI-18	chr2:26623669-26623732	AluYb8	AAAATCAGTTCTTCC	TTAAAA	CTCAAAAAAAAAAAAAAAAAAAAAAAAAAAAAATCAGTT

AI-19	chr9:37594172-37594310	AluY	AAGAAGTAGATATGG	TAA	CTCCAAAAAAAAAAAAAAGAAG

AI-20	chr6:99872263-99872632	-	-	-	-

AI-21	chr2:145175223-145175483	-	-	-	-

In addition to the Alu sequence itself, the genomic sequence adjacent to the recent Alu insertions encompass at least two typical sequences. As a hallmark of a recent retrotransposition event, the sequences immediately flanking the Alu elements corresponded to short direct repeats, ranging from 4-17 nucleotides. The insertion mechanism generates direct target site duplications (TSDs) flanking the newly inserted element. These TSDs have variable length and are highly suggestive of LINE mediated endonucleolytic cleavage [12,38]. Such short direct repeats, also called AT-rich target site duplications, were present in 19 of the sequence-verified genomic loci (Table 2).

### Methylation status of recent Alu elements

All sequences generated in our previous studies, encompassing Alu elements and their 5' flanking sequences, were derived from bisulfite converted genomic DNA [31,32]. Due to the high frequency of C-to-T transitions in CpG dinucleotides of Alu repeats caused by deamination of the methylated cytosines, in the absence of a reference genomic sequence, one cannot determine the methylation status of a novel Alu insertion by this method. Hence, to examine the methylation pattern of the newly integrated Alu elements, we aligned the sequences generated in this study for nineteen of such Alu elements with their bisulfite converted sequences from our previous studies [31,32] (Additional File 3, Figure S1). Our results showed that the recently inserted Alu elements are heavily methylated, with an average methylation level of 90.7%; this is similar to the average methylation level observed for evolutionarily young non-polymorphic Alu elements [31,32]. We further examined the methylation status of the two important promoter regions inside the Alu elements, the A and B boxes. Alu elements have a bipartite structure, which is similar to that of tRNA elements. It has been shown that the A box is responsible for determining the strength of the Pol III promoter and the B box is important in enabling transcription [30,39]. Also, deletion of the B box sequence completely abolished transcription of the elements, while deletion of the A box reduced the efficiency of transcription [40]. In almost all cases, these promoter sequences were methylated (Additional File 3, Figure S1). This result suggests that transcription of most newly inserted Alu elements is suppressed by DNA methylation.

**Figure 3:**
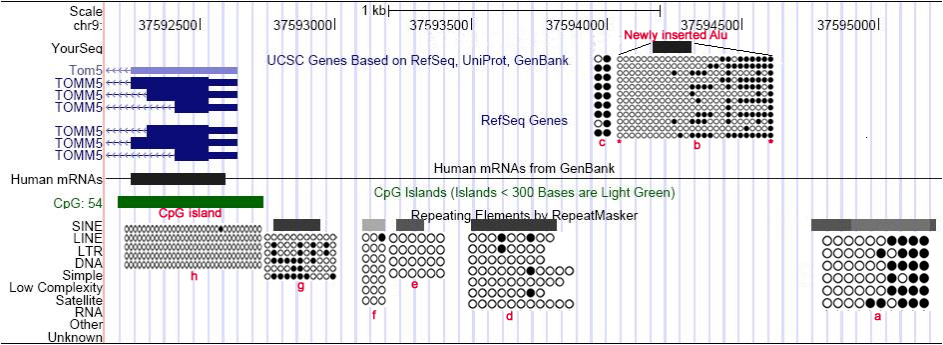
**Bisulfite PCR cloning and sequencing to validate methylation status of an unmethylated Alu insertion (chr9:37594172-37594310)**. Asterisk indicates the CpG dinucleotides that are flanking the Alu element; the scheme shows the relative location of TOMM5 and the CpG island in relation to the Alu AI19 insertion (USCS Genome Bioinformatics). a) methylation status of a downstream AluJo (sequence coordinates: chr9:37594745-37595002) near the newly inserted Alu element; b) newly inserted Alu element and its methylation status; c) methylation status of 2 CpGs upstream of the newly inserted Alu; d), e), f), and g) methylation statuses of 3 Alu repeats and 1 MIR element localized between the newly inserted Alu and the CpG island, respectively, AluSx, AluJo, MIRb, and AluSx; h) methylation status of the 5'end of a CpG island located 1,576 bp (sequence coordinates: chr9:37592324-37592701) upstream from the newly inserted Alu element. Note that the TOMM5 transcription unit is in opposite orientation to that of the newly inserted Alu element. The methylation levels for a, b, c, d, e, f, g, and h were 40%, 33.7%, 79.1%, 4.2%, 0%, 3%, 32%, and 0.6%, respectively.

Interestingly, we found one Alu element at chr9:37594172-37594310 with a completely unmethylated 5'-end (AI19, Additional File 3, Figure S1). Since amongst all Alu elements chosen for verification this was the only element found to be unmethylated, and also because only two bisulfite sequence reads had been previously generated for this element [31,32], we designed bisulfite PCR primers to amplify the entire Alu element including the two flanking CpG dinucleotides (Figure 3). Indeed, the 5'-end of this newly inserted Alu element was found to be completely unmethylated while its 3'-end exhibited some degree of DNA methylation. It is noteworthy that the 5'-flanking CpG site was completely unmethylated, and the 3'-flanking CpG site was completely methylated. Importantly, the 5' terminal nucleotide of this newly inserted Alu element mapped 1,576 bp downstream from a CpG island and 1,674 bp downstream from the transcription start site of the TOMM5 gene. This result suggests that the methylation status of this Alu element is under the influence of the epigenetic environment surrounding its insertion site. Since this Alu insertion was found to be in homozygosity, i.e. it was present in the two homologous chromosomes, we were not able to investigate whether the Alu insertion exerted any influence on the methylation status of CpG dinucleotides flanking the Alu element. To confirm our hypothesis that the methylation of the Alu element is under the influence of the CpG island, we ascertained the methylation status of a fragment (chr9:37592324-37592701) corresponding to the 5' terminal 377 bp of the CpG island, and also of the AluJo element flanking the 3'end (chr9:37594745-37595002) of the newly identified Alu element that was partially methylated. Indeed, we found that this CpG island fragment was completely unmethylated while the AluJo sequence flanking the 3'end of the newly inserted Alu exhibited a methylation level of the order of 40%. Interestingly, this AluJo exhibited a pattern of methylation very similar to the pattern presented by the newly inserted Alu element (Figure 3). There are 3 Alu repeats and 1 MIR element localized between the newly inserted Alu and the CpG island. The methylation levels of these elements are indeed very low (Figure 3).

**Figure 4:**
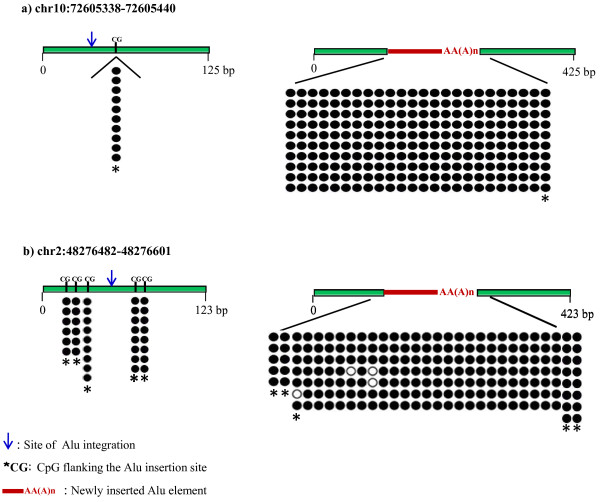
**Methylation statuses of two pairs of hemizygous alleles, i.e. before and after Alu insertions**. a) chr10:72605338-72605440 locus; b) chr2:48276482-48276601 locus. Schemes on the left side represent the allele not containing the Alu insertion, while the figure on the right side represents the allele in which the Alu element inserted. The blue arrow indicates the probable site of Alu integration; CG and asterisk indicate the CpG dinucleotides that are flanking the Alu element; red bar indicates the Alu element that was inserted.

We conducted similar analysis to two other genomic loci, chr10:72605338-72605440 and chr2:48276482-48276601, which were randomly chosen. The Alu insertions on these two loci were found to be in hemizygosity. This allowed us to compare the methylation status of the alleles with and without the Alu insertion (Figure 4). The sequencing results derived from bisulfite-PCR cloning demonstrated that both newly inserted Alu elements were indeed heavily methylated, as anticipated based upon our previously generated high-throughput bisulfite sequencing data (AI10 and AI11, Additional File 3, Figure S1). In addition, for the two genomic loci examined, there was no methylation difference between the alleles with and without the Alu insertion in the two homologous chromosomes, nor was there a difference in the methylation statuses of the CpG dinucleotides flanking the chr2:48276482-48276601 Alu insertion site. Furthermore, the CpG dinucleotide that is immediately downstream of the chr10:72605338-72605440 Alu insertion site was also found to be methylated. Due to the low CpG density of its 5' flanking genomic sequence, no methylation data were derived for the region upstream of chr10:72605338-72605440. To identify methylation differences among samples, we calculated the methylation level of all mapped Alu elements, and also that of the structural variants present in the 19 loci verified. This analysis revealed no methylation differences among tissues (Additional File 4, Table S3).

## Discussion

Recent studies demonstrated that major structural variants in the human genome are derived from retrotransposons, Alu elements in particular [16,41]. Due to the extensive sequence homology that exists among young Alu repeats, the identification of such structural variants remains a challenging task. To date, a total of 2,558 polymorphic Alu retrotransposons have been reported to occur in human populations, 1,763 of which have been deposited in dbSNP and 795 in dbRIP. In this study we implemented a computational pipeline to identify recent Alu insertions, and examined the methylation status of the newly inserted Alu retrotransposons and their flanking sequences. At the time we developed this strategy the Genome Sequencer FLX System was the most suitable alternative available, given the greater length of the sequence reads that it generates, and the fact that sequences would encompass an Alu repeat and would be derived from bisulfite-converted genomic DNA. Altogether, the longer reads generated with the FLX System greatly facilitated their mapping back to the reference genome sequence. Notwithstanding this advantage, however, we anticipate that our approach may be adapted to take advantage of competing next generation sequencing platforms that have a higher throughput and that can now generate sufficiently long sequence reads.

Using this strategy a total of 327 putative Alu elements were identified. We found that 42.8% of their insertion sites fell within intronic regions, while one integration site mapped to the 3'-UTR of the *TOMM40 *gene. TOMM40 is a component of the preprotein translocase complex of the outer mitochondrial membrane, which consists of at least 7 different proteins (TOMM5, TOMM6, TOMM7, TOMM20, TOMM22, TOMM40, and TOMM70). These results are consistent with previous studies indicating that Alu retrotransposons tend to be inserted within intragenic regions [1,42].

Out of the twenty-one insertion events that were randomly selected for validation analysis, nineteen were successfully verified. A limitation of the Alu-anchored bisulfite PCR approach that needs to be acknowledged is the fact that only 5' flanking sequences are obtained. The right arm of the Alu retrotransposons and their 3' flanking sequences are not represented in the sequence reads that are generated. Hence, in order to design primers for the validation experiments, we used the reference sequence of the human genome as source of putative 3'-flanking sequences for the Alu insertions. Accordingly, it is possible that the two cases that could not be verified may have been caused by the utilization of an incorrect 3' flanking sequence for primer design. Notwithstanding this limitation, the lowest estimated accuracy for the analysis pipeline that we have implemented in this study for the identification of *de novo *Alu retrotransposition events would be of 90.5% (19/21).

The sequence features (TSD, TT/AAAA cleavage sequence, and A-rich Alu tail) that are typically observed in newly inserted Alu elements constitute hallmarks of retrotransposition [5,10,36,37]. Indeed, further analysis of the aforementioned nineteen PCR-cloned Alu elements and flanking sequences revealed the presence of both the TSD and TT/AAAA sites. Alu A-tails seem to be an important factor to enable Alu element retrotransposition [4,35]. Roy-Engel et al. reported that the average A-tail length of active Alu elements is 26 [35]. Consistent to their finding, the Alu A-tail sizes of the Alu elements described in this study ranged from 11-45 with an average of 29 bp.

Most cancer genomes are characterized by localized hypermethylation as well as by global hypomethylation [43,44]. This hypomethylation process may enable transcription and *de novo *retrotransposition of Alu elements which, in turn, may lead to genome instability [45]. Our previous study demonstrated that the methylation level of Alu elements decreased in ependymomas, and most significantly in recurrent tumors [31]. To examine whether some Alu insertions represented somatic events limited to recurrent ependymomas, which could have occurred in consequence of the loss of DNA methylation, we generated and compared PCR products from ten genomic loci in primary and in recurrent tumors derived from one individual. The same results were obtained in all ten genomic loci. In addition, five of the ten Alu insertions were also found in other individuals. These results suggested that such validated Alu insertions most likely represent germ-line rather than somatic events.

In this study, in addition to identifying structural variants in the genome of 6 individuals, we investigated epigenetic variations that might result from *de novo *retrotransponsition events. The Alu elements identified in this study were heavily methylated, as it was previously shown by high-throughput bisulfite sequencing and herein validated by cloning and sequencing analyses. The analysis of methylation throughout the mapped Alus and among the 19 loci verified revealed that there were no methylation differences among tissues (Additional File 4, Table S3). This result indicates that at least by the time these DNA samples were obtained most of the newly inserted Alu elements were already transcriptionally repressed. This finding is further supported by the fact that the promoters of the Alu elements, i.e. their A and B boxes, were found to be methylated. However, there was one exception. We found an Alu insertion that was partially unmethylated (chr9:37594172-37594310). Interestingly, the insertion of this Alu element occurred 1,576 bp downstream from a CpG island and 1,674 bp downstream from the transcription start site of TOMM5, a gene encoding the translocase of the outer mitochondrial membrane 5. With a completely unmethylated promoter (both the A box and the B box were unmethylated), it is conceivable that this Alu element may have remained transcriptionally active and hence have served as source for additional retrotransposition events. Another interesting finding was that a CpG island that is upstream of the element - i.e., that of TOMM5 - may be influencing the methylation pattern of this Alu repeat. Indeed, the methylation status of the CpG island was similar to that of the 5' end sequences of this Alu repeat, i.e. both were unmethylated. It would be interesting to explore the functional impact of this particular Alu on the nearby TOMM5 gene. Additionally, 3 Alu repeats and 1 MIR element that are localized between the newly inserted Alu repeat and the CpG island were found to exhibit very low methylation levels. Such striking pattern of DNA methylation may indeed be an indication of the influence exerted by the adjacent CpG island. It is also possible that other epigenetic factors might be affecting the methylation statuses of these Alu elements, such as nucleosome positioning. Two previous studies have reported the influence of nucleosome positioning, within and around Alu element, in Alu activity [46,47]. Accordingly, it is noteworthy that an AluJo that is localized downstream of this newly inserted Alu exhibits a similar pattern of DNA methylation, i.e. its 5' half is unmethylated while its 3' half is methylated. In our previous study [32], we found that genomic localization has a profound impact on Alu methylation status. In this study, the identification of both methylated and unmethylated Alu elements provided additional support to there being a positional effect on Alu methylation. Last, but not least, it is noteworthy that two of the novel Alu insertions herein reported map within or near genes encoding members of the preprotein translocase complex of the outer mitochondrial membrane, namely TOMM40 and TOMM5, respectively. It is conceivable that given their housekeeping function and ubiquitous expression pattern, hence commonly open chromatin status, these genes may be more vulnerable to uptake *de novo *retrotransposition events.

To explore the epigenetic impact of Alu insertion on adjacent genomic sequences, we examined the methylation statuses for two loci harboring hemizygous insertions, and - in one case - obtained the methylation patterns of CpG dinucleotides flanking the Alu insertion sites. Both alleles - irrespective of the presence of an inserted element - were found to be heavily methylated, and no significant epigenetic variation was observed in association with the presence of the additional Alu element.

## Conclusions

In this work we have identified a few novel Alu insertions sites. We used DNA samples from normal and from tumor tissues, but the data obtained did not show any tissue preference for these insertions. More studies are highly desired to further scrutinize the functional aspects of structural variants in the human genome, including epigenetic variations that might arise in consequence of a *de novo *retrotransposition event.

## Methods

### High-throughput bisulfite sequencing datasets for Alu elements

The high-throughput bisulfite sequencing data were derived from Alu-anchored bisulfite PCR libraries derived from tissues samples, including a normal cerebellum, a normal 4^th ^ventricle lining, two primary non-aggressive, two primary aggressive and two recurrent ependymomas [31,32]. Briefly, genomic DNA is first digested with AluI restriction enzyme, ligated to adaptors and then subjected to bisulfite treatment. Bisulfite treated DNA is amplified with adaptor and Alu-specific primers, the latter targeting a large pool of CpG-rich Alu elements. Thus, each PCR product contains the 5'end of an Alu element and its (most often) unique flanking genomic sequence, which makes it possible for each sequence to be unambiguously mapped to the reference human genome. Primary non-aggressive ependymomas are defined as primary tumors from patient free of disease progression for more than 4 years and primary aggressive ones are defined as primary tumors from patients with recurrent disease within 3 years or deceased of disease.

### Computational pipeline for the identification of recent Alu insertions

To identify putatively recent Alu insertions, sequence reads rejected in previous studies were selected. Briefly, after removal of primer and adaptor sequences, sequences greater than 40 bp were aligned to the *in silico *bisulfite converted reference genome using multiple cycles of MegaBLAST. The word size used in Megablast was set to 100 for the first cycle, it was decreased by 20 for every cycle thereafter until the last, for which the minimum length of best perfect match was set to 40. In addition, the identity percentage cutoff for a significant alignment was set to be 100 for the last cycle and 95 for all other cycles of Megablast [32]. The sequence reads that mapped to genomic loci within 10 bp from an Alu element were considered as a putative recent Alu insertion.

### PCR, cloning, and sequencing

For PCR primer design, the original (not bisulfite converted) DNA sequences flanking the predicted Alu insertion sites were extracted from the UCSC reference human genome, based on their genomic coordinates [48]. PCR primers were designed in the region surrounding the Alu insertion sites. PCR reactions were performed using HotStartTaq^R ^Plus Master Mix from QIAGEN. Each reaction was prepared as follows: 12.5 μL of HotStartTaq^R ^mix, 30 ng of DNA, 14 μM of each primer, and enough water for 25 μL. The PCR reactions were performed on a MJ Research machine (model PTC 225). Reactions were subjected to an initial activation step of 95°C for 15 min, then by a denaturation step of 94°C for 1 min, followed by 40 cycles of 1 min at 94°C, 30 s at optimal annealing temperature, and 40 s at 72°C, followed by a final extension step of 10 min at 72°C. PCR product annealing temperatures (Tm) and primers used on each reaction are listed on Additional File 5, Table S4. After reactions were completed the amplified fragments were separated using 1.5% agarose gel electrophoresis that was stained with ethidium bromide and visualized using UV fluorescence system. Running was carried out until a good separation of bands was obtained. After separation in the 1.5% agarose gel the bands were excised off the gel and purified using a gel purification kit from QIAGEN, QIAquick^R ^PCR Purification Kit. The purified PCR products were cloned using the TOPO TA Cloning^R ^System from Invitrogen. Sequencing reactions for individual colonies were conducted at the Sequencing Core Facility of the Children's Memorial Research Center of Northwestern University's Feinberg School of Medicine.

### Bisulfite PCR

Bisulfite conversion of genomic DNA was performed with EZ DNA Methylation Gold kit (Zymo Research Corporation, Irvine, CA) following the manufacturer's instructions. 300 ng of genomic DNA was treated and eluted with 10 μL of elution solution. After this step, DNA from the chr10:72275361-72275449 genomic locus was amplified using the pair of primers: 5'- GGA TTA AGT TTT TTT TTT GTT T -3' and 5'- CTA CAA AAA AAA ATA ACT CAT A -3'; the chr2:48129974-48130105 genomic locus was amplified using the pair of primers: 5'- CCT TAC CAT TTA AAA ATA AAA AAT CAA -3' and 5'- GTT TAA GAT TTA AAG GAA TGA GTT AG -3'. PCR reactions were prepared using the same reagents and conditions described above. The PCR program used was: activation step of 95°C for 15 min, then by a denaturation step of 94°C for 1 min, followed by 40 cycles of 1 min at 94°C, 30 s at optimal annealing temperature (49°C for locus chr10:72605338-72605440 and 42°C for locus chr2:48276482-48276601), and 40 s at 72°C, followed by a final extension step of 10 min at 72°C. PCR amplified fragments were separated in 1.5% agarose and excised from it as described above.

To amplify the 5'end of the CpG island and the sequence flanking the 3' end of the Alu element (AI19 chr9:37594172-37594310) we used nested PCR. The primers used for the CpG island were: external primers (TOMM5-eFor 5'- AAG TTG GGA GAA TTA GGA TGA TT -3' and TOMM5-eRev 5'- CTA ATT TTT ATA TAA CAA ATA TTA TTA AAA ACA AC -3'), internal primers (TOMM5-iFor 5'- GTA TTT TTA GAG TTA AGG GGT GT -3' and TOMM5-iRev 5'- CAC TTC AAA TCA ACT AAA TCA AAA C -3'). The primers used for the 3'Alu flanking sequence were: external primers (ch9Down-eFor 5'- TTT GTA GTG ATG TTG AAA GTA GTA AGA- 3' and ch9Down-eRev 5'- AAT ATA TAC CTT CCC TTT CCA ACT -3'), internal primers (ch9Down-iFor 5'- TTT ATT TTA GAT TGA GTT TTG TTT TGT -3' and ch9Down-iRev 5'- CTT AAA CCC AAA AAT ATA AAA TTA CAA TAC -3'). The PCR program used was the same mentioned above, the TM temperatures were 51°C for external primers and 50°C for internal primers.

### Alu classification

As it was mentioned above, the initial data used for this study were derived from Alu-anchored bisulfite PCR libraries. These libraries were constructed using primers specially designed to target the most recent and active Alu elements, which are the ones that belong to AluY family. To classify the Alu insertions verified in this study within the AluY family, we aligned the sequences generated in this study with those contained in the UCSC Genome browser. From this alignment, the element with the highest score was used to classify a newly identified Alu insertion into the AluY family.

## Authors' contributions

AA designed the study, carried out the experiments, and helped to draft the manuscript. MW participated in the data analysis. MFB participated in the design of the study and helped with the experiments. HX contributed to the conception of the study, participated in the design of the study, and helped to draft the manuscript. MBS contributed to the conception of the study, responsible for coordination of the study, and was responsible for the final editing and revision of the manuscript. All Authors read and approved the final manuscript.

## Supplementary Material

Additional file 1 **Table S1**. Putatively recent Alu insertions. Alu insertions identified in eight Alu bisulfite PCR libraries. Click here for file

Additional file 2 **Table S2**. Clusters of putative Alu insertions. Alu insertions and their gene annotation. Click here for file

Additional file 3 **Figure S1**. Methylation pattern of recent Alu insertions. To determine the methylation status, the sequences corresponding to the first half of Alu elements plus its 5' flanking regions [31,32] were aligned to the Alu element sequences generated in this study. NC1: Normal cerebellum and NC2: normal 4^th ^ventricle lining tissue; PA1, PA2, PA3, PA4, and PA5: primary ependymoma tumor; RL: ependymoma tumor relapsed from PA3. Click here for file

Additional file 4 **Table S3**. Alu methylation level. Methylation levels of mapped and 19 non-mapped Alu elements. Click here for file

Additional file 5 **Table S4**. Validation of identified Alu elements. Primers designed for Alu elements validation. Click here for file

## References

[B1] Lander ES Linton LM Birren B Nusbaum C Zody MC Baldwin J Devon K Dewar K Doyle M FitzHugh W Initial sequencing and analysis of the human genome Nature 2001 409 6822 860 921 10.1038/35057062 11237011

[B2] Paolella G Lucero MA Murphy MH Baralle FE The Alu family repeat promoter has a tRNA-like bipartite structure EMBO J 1983 2 5 691 696 16453450 10.1002/j.1460-2075.1983.tb01486.x PMC555171

[B3] Weiner AM An abundant cytoplasmic 7 S RNA is complementary to the dominant interspersed middle repetitive DNA sequence family in the human genome Cell 1980 22 1 Pt 1 209 218 6159101 10.1016/0092-8674(80)90169-5

[B4] Comeaux MS Roy-Engel AM Hedges DJ Deininger PL Diverse cis factors controlling Alu retrotransposition: what causes Alu elements to die? Genome Res 2009 19 4 545 555 10.1101/gr.089789.108 19273617 PMC2665774

[B5] Dewannieux M Esnault C Heidmann T LINE-mediated retrotransposition of marked Alu sequences Nat Genet 2003 35 1 41 48 10.1038/ng1223 12897783

[B6] Liu GE Alkan C Jiang L Zhao S Eichler EE Comparative analysis of Alu repeats in primate genomes Genome Res 2009 19 5 876 885 10.1101/gr.083972.108 19411604 PMC2675976

[B7] Batzer MA Deininger PL Hellmann-Blumberg U Jurka J Labuda D Rubin CM Schmid CW Zietkiewicz E Zuckerkandl E Standardized nomenclature for Alu repeats J Mol Evol 1996 42 1 3 6 10.1007/BF00163204 8576960

[B8] Chen JM Stenson PD Cooper DN Ferec C A systematic analysis of LINE-1 endonuclease-dependent retrotranspositional events causing human genetic disease Hum Genet 2005 117 5 411 427 10.1007/s00439-005-1321-0 15983781

[B9] Batzer MA Deininger PL Alu repeats and human genomic diversity Nat Rev Genet 2002 3 5 370 379 10.1038/nrg798 11988762

[B10] Roy AM Carroll ML Kass DH Nguyen SV Salem AH Batzer MA Deininger PL Recently integrated human Alu repeats: finding needles in the haystack Genetica 1999 107 1-3 149 161 10952208

[B11] Batzer MA Kilroy GE Richard PE Shaikh TH Desselle TD Hoppens CL Deininger PL Structure and variability of recently inserted Alu family members Nucleic Acids Res 1990 18 23 6793 6798 10.1093/nar/18.23.6793 2175877 PMC332733

[B12] Bennett EA Keller H Mills RE Schmidt S Moran JV Weichenrieder O Devine SE Active Alu retrotransposons in the human genome Genome Res 2008 18 12 1875 1883 10.1101/gr.081737.108 18836035 PMC2593586

[B13] Iskow RC McCabe MT Mills RE Torene S Pittard WS Neuwald AF Van Meir EG Vertino PM Devine SE Natural mutagenesis of human genomes by endogenous retrotransposons Cell 2010 141 7 1253 1261 10.1016/j.cell.2010.05.020 20603005 PMC2943760

[B14] Chow JC Ciaudo C Fazzari MJ Mise N Servant N Glass JL Attreed M Avner P Wutz A Barillot E LINE-1 activity in facultative heterochromatin formation during X chromosome inactivation Cell 2010 141 6 956 969 10.1016/j.cell.2010.04.042 20550932

[B15] Beck CR Collier P Macfarlane C Malig M Kidd JM Eichler EE Badge RM Moran JV LINE-1 retrotransposition activity in human genomes Cell 2010 141 7 1159 1170 10.1016/j.cell.2010.05.021 20602998 PMC3013285

[B16] Xing J Zhang Y Han K Salem AH Sen SK Huff CD Zhou Q Kirkness EF Levy S Batzer MA Mobile elements create structural variation: analysis of a complete human genome Genome Res 2009 19 9 1516 1526 10.1101/gr.091827.109 19439515 PMC2752133

[B17] Deininger PL Batzer MA Alu repeats and human disease Mol Genet Metab 1999 67 3 183 193 10.1006/mgme.1999.2864 10381326

[B18] Kolomietz E Meyn MS Pandita A Squire JA The role of Alu repeat clusters as mediators of recurrent chromosomal aberrations in tumors "Genes, Chromosomes and Cancer" 2002 35 2 97 112 10.1002/gcc.10111 12203773

[B19] Roy-Engel AM El-Sawy M Farooq L Odom GL Perepelitsa-Belancio V Bruch H Oyeniran OO Deininger PL Human retroelements may introduce intragenic polyadenylation signals Cytogenet Genome Res 2005 110 1-4 365 371 10.1159/000084968 16093688

[B20] Callinan PA Batzer MA Retrotransposable elements and human disease Genome Dyn 2006 1 104 115 18724056 10.1159/000092503

[B21] Zemojtel T Kielbasa SM Arndt PF Chung HR Vingron M Methylation and deamination of CpGs generate p53-binding sites on a genomic scale Trends Genet 2009 25 2 63 66 10.1016/j.tig.2008.11.005 19101055

[B22] Sasaki T Nishihara H Hirakawa M Fujimura K Tanaka M Kokubo N Kimura-Yoshida C Matsuo I Sumiyama K Saitou N Possible involvement of SINEs in mammalian-specific brain formation Proc Natl Acad Sci USA 2008 105 11 4220 4225 10.1073/pnas.0709398105 18334644 PMC2393765

[B23] Hewitt SM Fraizer GC Saunders GF Transcriptional silencer of the Wilms' tumor gene WT1 contains an Alu repeat J Biol Chem 1995 270 30 17908 17912 10.1074/jbc.270.30.17908 7629096

[B24] Wu J Grindlay GJ Bushel P Mendelsohn L Allan M Negative regulation of the human epsilon-globin gene by transcriptional interference: role of an Alu repetitive element Mol Cell Biol 1990 10 3 1209 1216 2304465 10.1128/mcb.10.3.1209-1216.1990 PMC360999

[B25] Mariner PD Walters RD Espinoza CA Drullinger LF Wagner SD Kugel JF Goodrich JA Human Alu RNA is a modular transacting repressor of mRNA transcription during heat shock Mol Cell 2008 29 4 499 509 10.1016/j.molcel.2007.12.013 18313387

[B26] Chu WM Ballard R Carpick BW Williams BR Schmid CW Potential Alu function: regulation of the activity of double-stranded RNA-activated kinase PKR Mol Cell Biol 1998 18 1 58 68 9418853 10.1128/mcb.18.1.58 PMC121451

[B27] Tufarelli C Stanley JA Garrick D Sharpe JA Ayyub H Wood WG Higgs DR Transcription of antisense RNA leading to gene silencing and methylation as a novel cause of human genetic disease Nat Genet 2003 34 2 157 165 10.1038/ng1157 12730694

[B28] Yoder JA Walsh CP Bestor TH Cytosine methylation and the ecology of intragenomic parasites Trends Genet 1997 13 8 335 340 10.1016/S0168-9525(97)01181-5 9260521

[B29] Rodriguez J Vives L Jorda M Morales C Munoz M Vendrell E Peinado MA Genome-wide tracking of unmethylated DNA Alu repeats in normal and cancer cells Nucleic Acids Res 2008 36 3 770 784 18084025 10.1093/nar/gkm1105 PMC2241897

[B30] Kochanek S Renz D Doerfler W Transcriptional silencing of human Alu sequences and inhibition of protein binding in the box B regulatory elements by 5'-CG-3' methylation FEBS Lett 1995 360 2 115 120 10.1016/0014-5793(95)00068-K 7875314

[B31] Xie H Wang M Bonaldo Mde F Rajaram V Stellpflug W Smith C Arndt K Goldman S Tomita T Soares MB Epigenomic analysis of Alu repeats in human ependymomas Proc Natl Acad Sci USA 2010 107 15 6952 6957 10.1073/pnas.0913836107 20351280 PMC2872440

[B32] Xie H Wang M Bonaldo Mde F Smith C Rajaram V Goldman S Tomita T Soares MB High-throughput sequence-based epigenomic analysis of Alu repeats in human cerebellum Nucleic Acids Res 2009 37 13 4331 4340 10.1093/nar/gkp393 19458156 PMC2715246

[B33] Sherry ST Ward MH Kholodov M Baker J Phan L Smigielski EM Sirotkin K dbSNP: the NCBI database of genetic variation Nucleic Acids Res 2001 29 1 308 311 10.1093/nar/29.1.308 11125122 PMC29783

[B34] Wang J Song L Grover D Azrak S Batzer MA Liang P dbRIP: a highly integrated database of retrotransposon insertion polymorphisms in humans Hum Mutat 2006 27 4 323 329 10.1002/humu.20307 16511833 PMC1855216

[B35] Roy-Engel AM Salem AH Oyeniran OO Deininger L Hedges DJ Kilroy GE Batzer MA Deininger PL Active Alu element "A-tails": size does matter Genome Res 2002 12 9 1333 1344 10.1101/gr.384802 12213770 PMC186649

[B36] Feng Q Moran JV Kazazian HH Boeke JD Human L1 retrotransposon encodes a conserved endonuclease required for retrotransposition Cell 1996 87 5 905 916 10.1016/S0092-8674(00)81997-2 8945517

[B37] El-Sawy M Deininger P Tandem insertions of Alu elements Cytogenet Genome Res 2005 108 1-3 58 62 10.1159/000080802 15545716

[B38] Hagan CR Sheffield RF Rudin CM Human Alu element retrotransposition induced by genotoxic stress Nat Genet 2003 35 3 219 220 10.1038/ng1259 14578886

[B39] Liu WM Maraia RJ Rubin CM Schmid CW Alu transcripts: cytoplasmic localisation and regulation by DNA methylation Nucleic Acids Res 1994 22 6 1087 1095 10.1093/nar/22.6.1087 7512262 PMC307934

[B40] Perez-Stable C Shen CK Competitive and cooperative functioning of the anterior and posterior promoter elements of an Alu family repeat Mol Cell Biol 1986 6 6 2041 2052 3023916 10.1128/mcb.6.6.2041-2052.1986 PMC367744

[B41] Lupski JR Retrotransposition and structural variation in the human genome Cell 2010 141 7 1110 1112 10.1016/j.cell.2010.06.014 20602993

[B42] Grover D Mukerji M Bhatnagar P Kannan K Brahmachari SK Alu repeat analysis in the complete human genome: trends and variations with respect to genomic composition Bioinformatics 2004 20 6 813 817 10.1093/bioinformatics/bth005 14751968

[B43] Deng G Nguyen A Tanaka H Matsuzaki K Bell I Mehta KR Terdiman JP Waldman FM Kakar S Gum J Regional hypermethylation and global hypomethylation are associated with altered chromatin conformation and histone acetylation in colorectal cancer Int J Cancer 2006 118 12 2999 3005 10.1002/ijc.21740 16425274

[B44] Roman-Gomez J Jimenez-Velasco A Agirre X Castillejo JA Navarro G Garate L Jose-Eneriz ES Cordeu L Barrios M Prosper F Promoter hypermethylation and global hypomethylation are independent epigenetic events in lymphoid leukemogenesis with opposing effects on clinical outcome Leukemia 2006 20 8 1445 1448 10.1038/sj.leu.2404257 16688225

[B45] Daskalos A Nikolaidis G Xinarianos G Savvari P Cassidy A Zakopoulou R Kotsinas A Gorgoulis V Field JK Liloglou T Hypomethylation of retrotransposable elements correlates with genomic instability in non-small cell lung cancer Int J Cancer 2009 124 1 81 87 10.1002/ijc.23849 18823011

[B46] Englander EW Howard BH Nucleosome positioning by human Alu elements in chromatin J Biol Chem 1995 270 17 10091 10096 10.1074/jbc.270.17.10091 7730313

[B47] Tanaka Y Yamashita R Suzuki Y Nakai K Effects of Alu elements on global nucleosome positioning in the human genome BMC Genomics 11 309 10.1186/1471-2164-11-309 PMC2878307 20478020

[B48] Kuhn RM Karolchik D Zweig AS Trumbower H Thomas DJ Thakkapallayil A Sugnet CW Stanke M Smith KE Siepel A The UCSC genome browser database: update 2007 Nucleic Acids Res 2007 35 Database D668 673 10.1093/nar/gkl928 PMC1669757 17142222

