# Supplementary figures and images for: Genetic and epigenetic variations contributed by Alu retrotransposition
Source: BMC Genomics. 2011 Dec 20;12:617. doi: 10.1186/1471-2164-12-617 (PMC3272032; doi:10.1186/1471-2164-12-617)

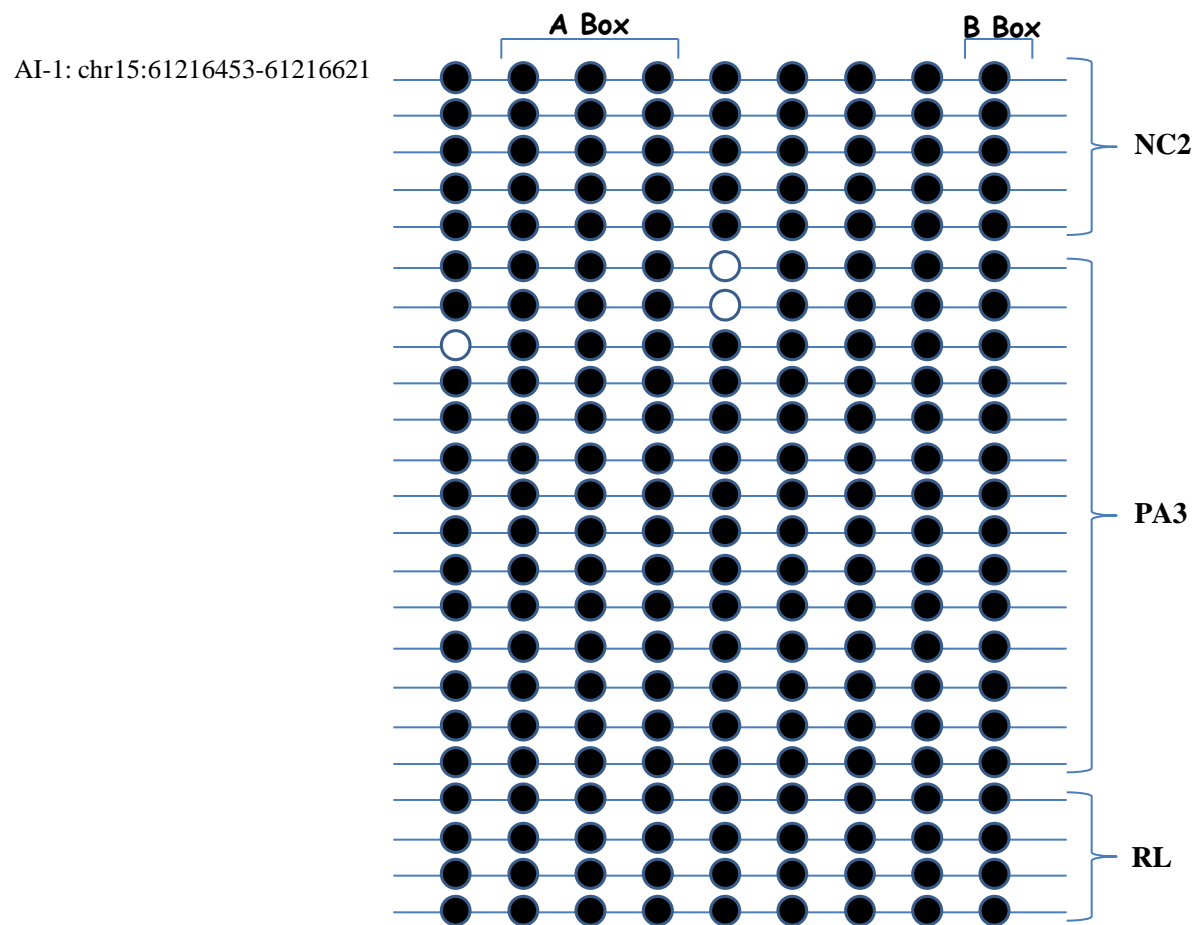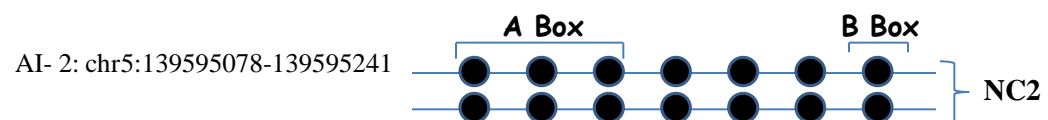

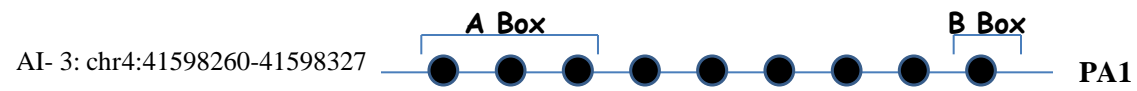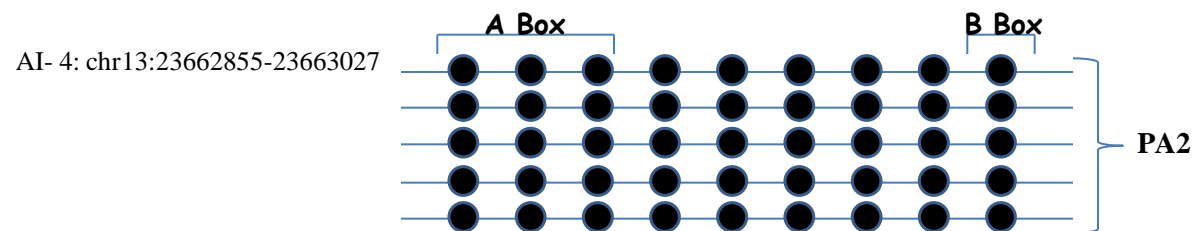

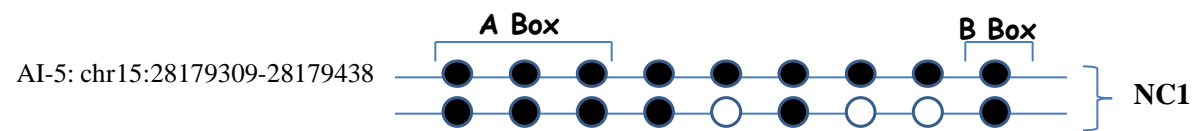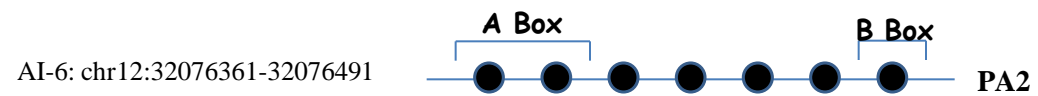

AI-7: chr11:130675880-130675924

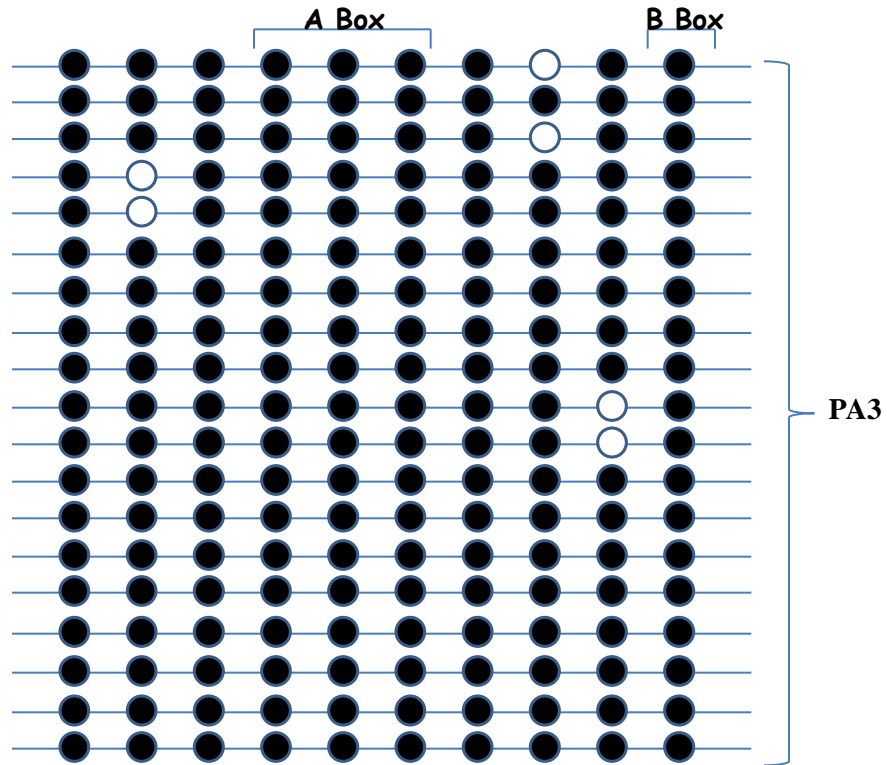

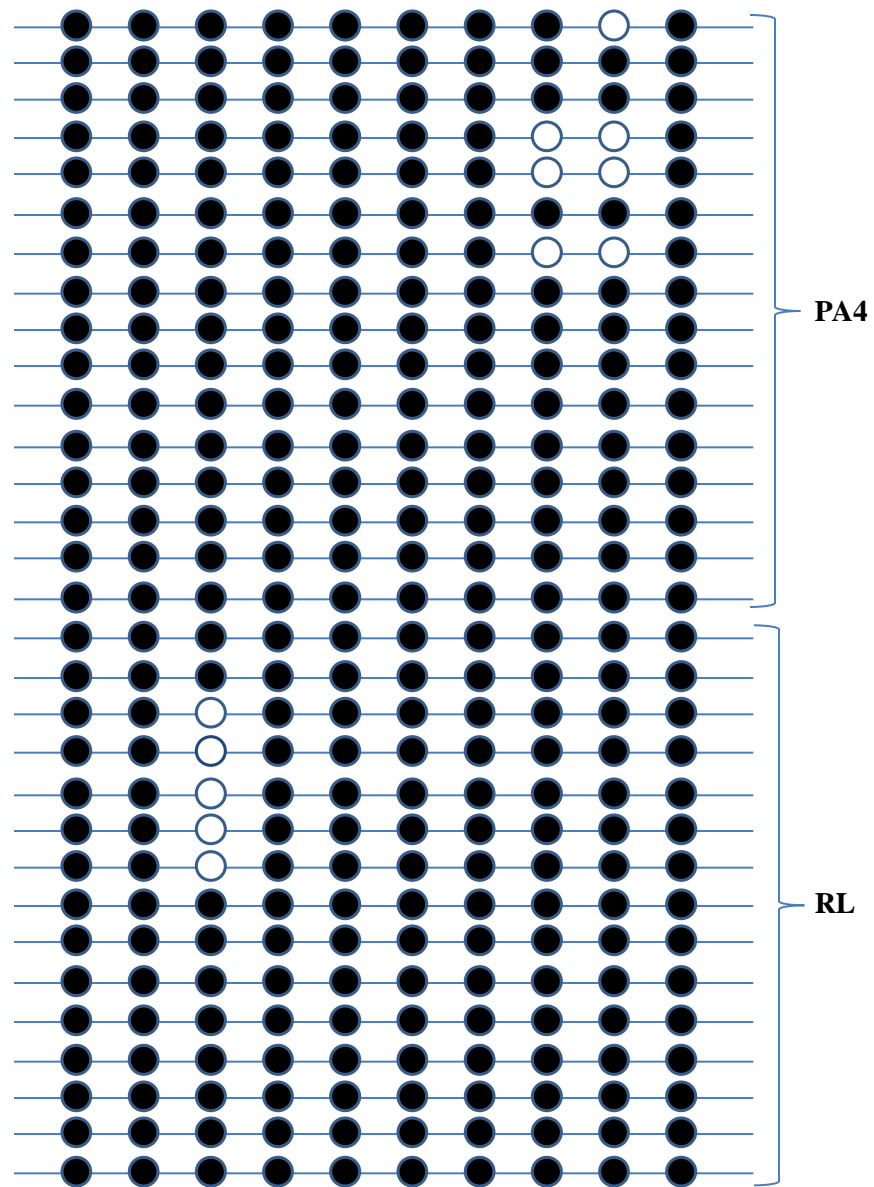

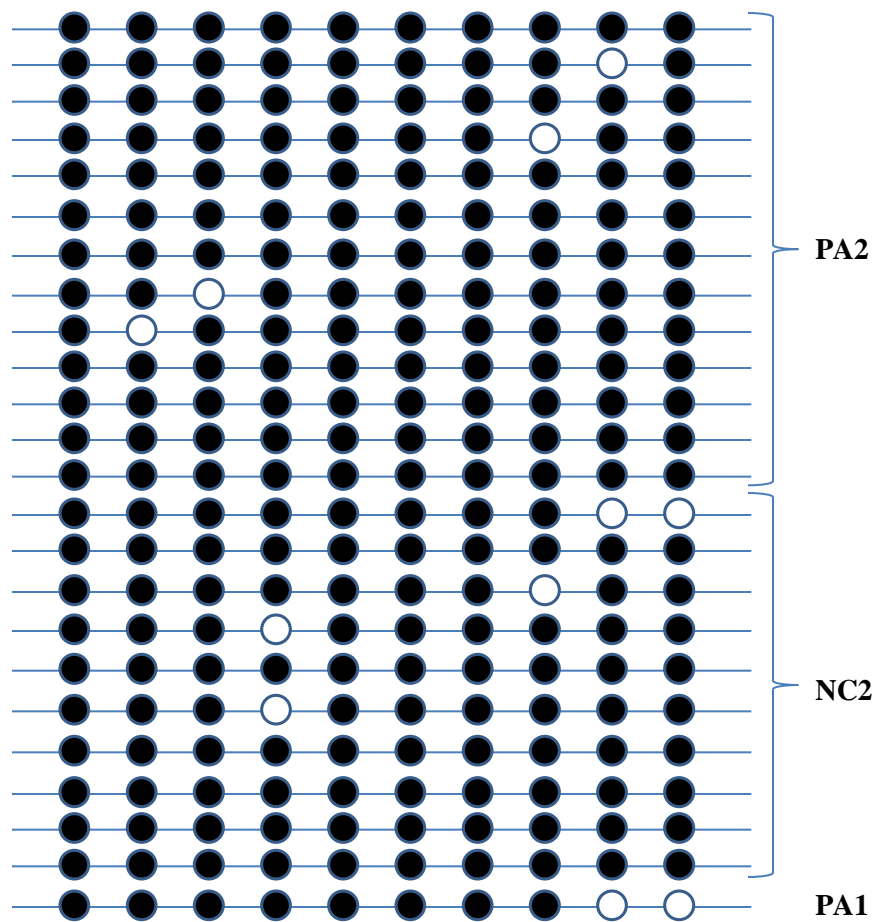

AI-8: chr5:141758572-141758694

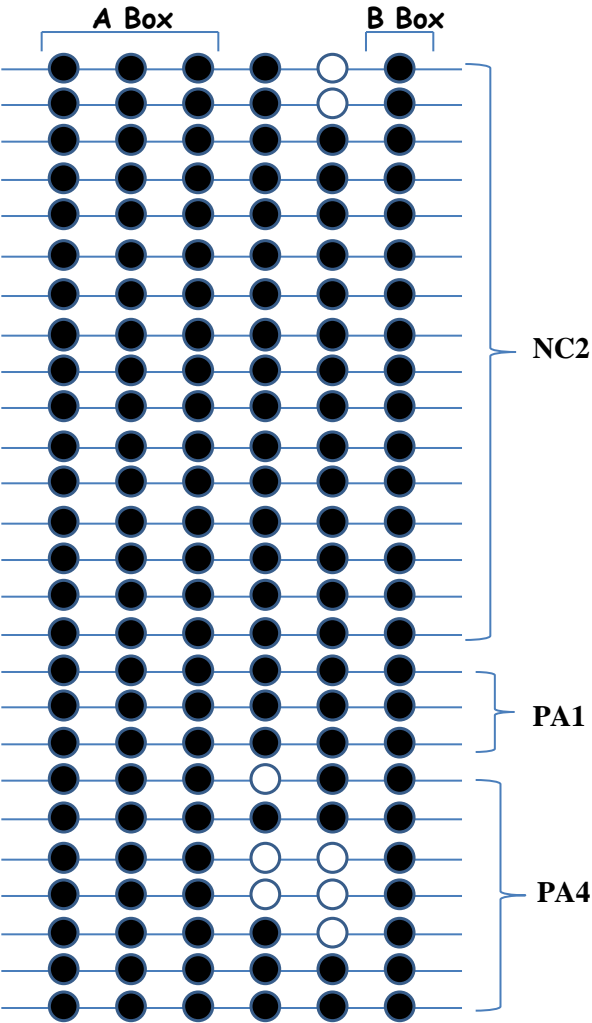

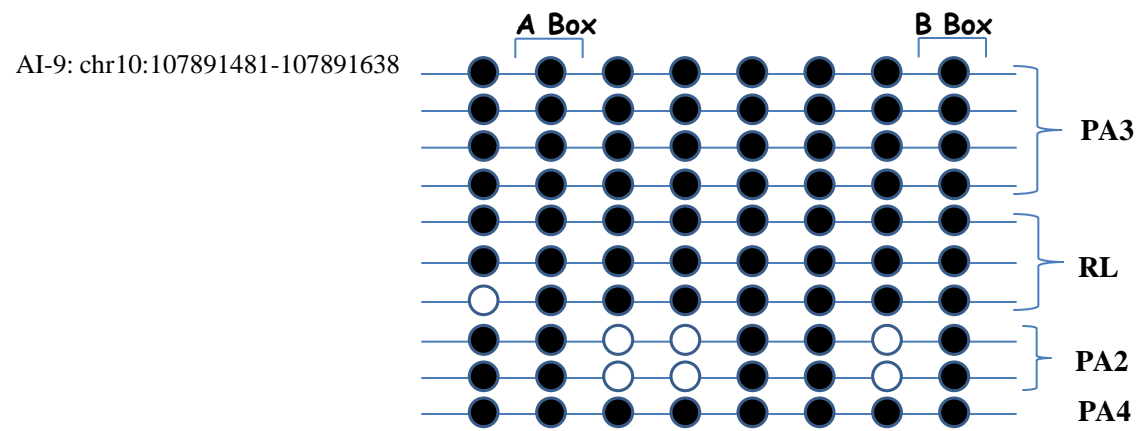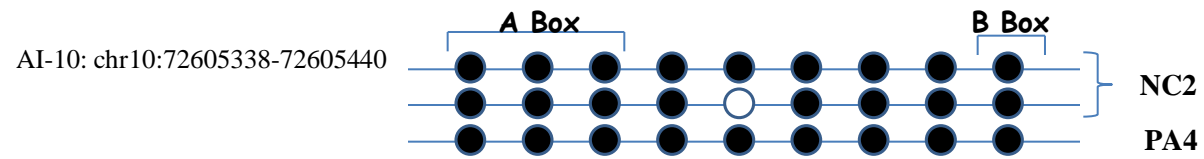

AI-11:chr2:48276482-48276601

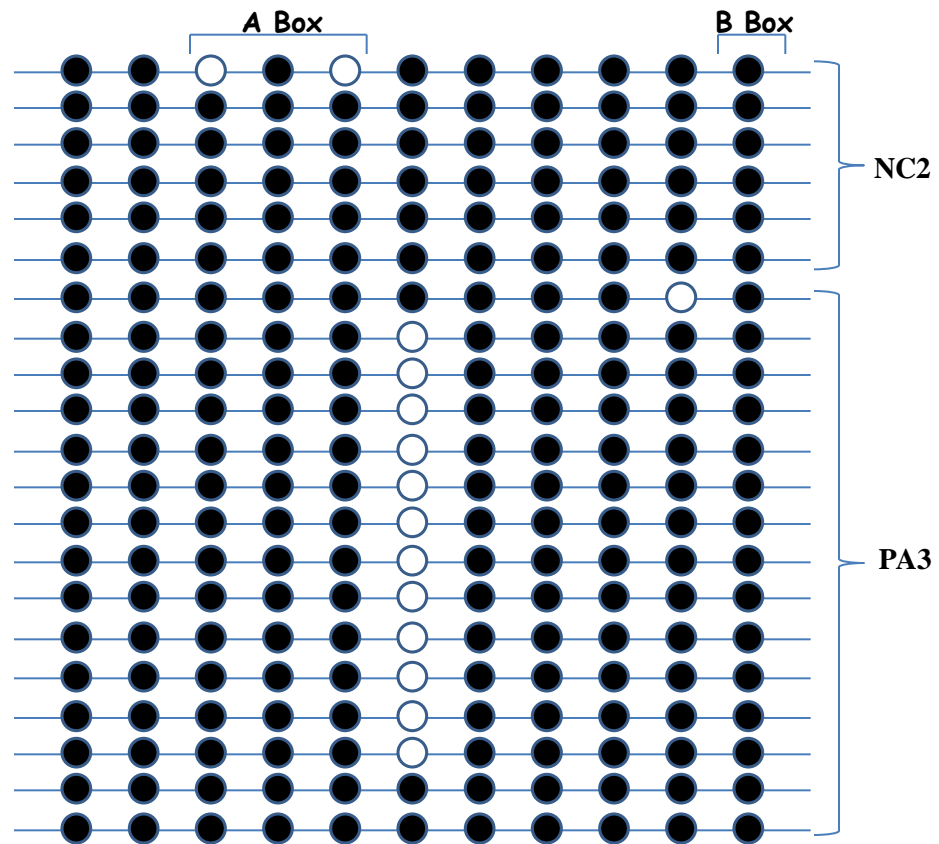

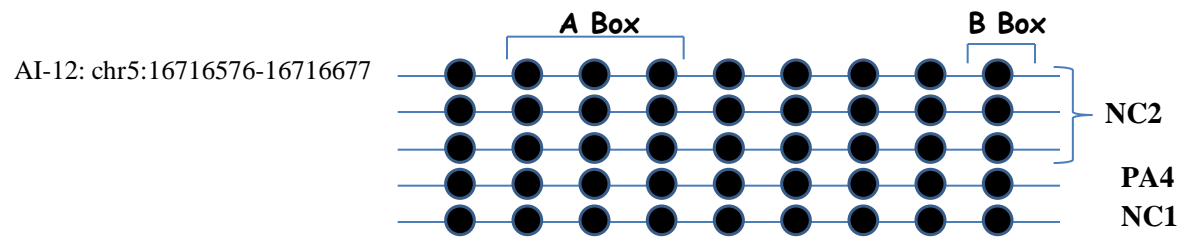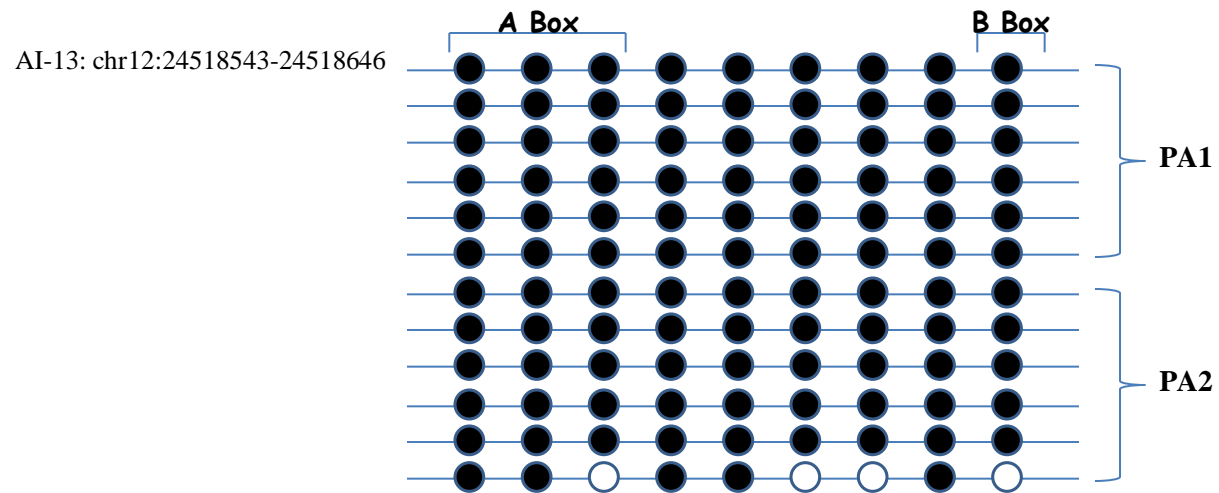

AI-14: chr6:57403535-57403610

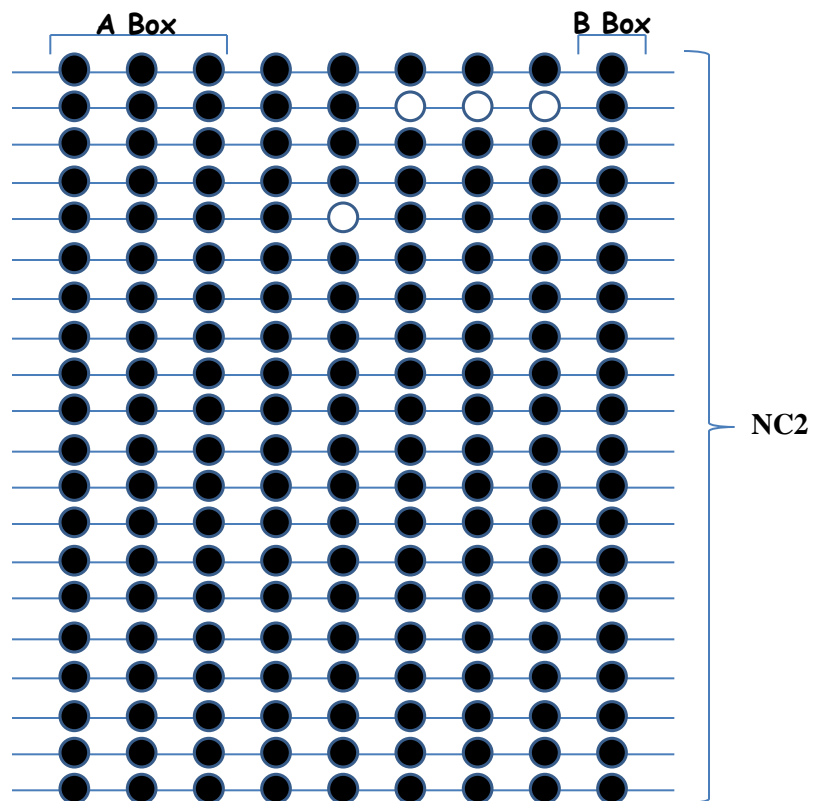

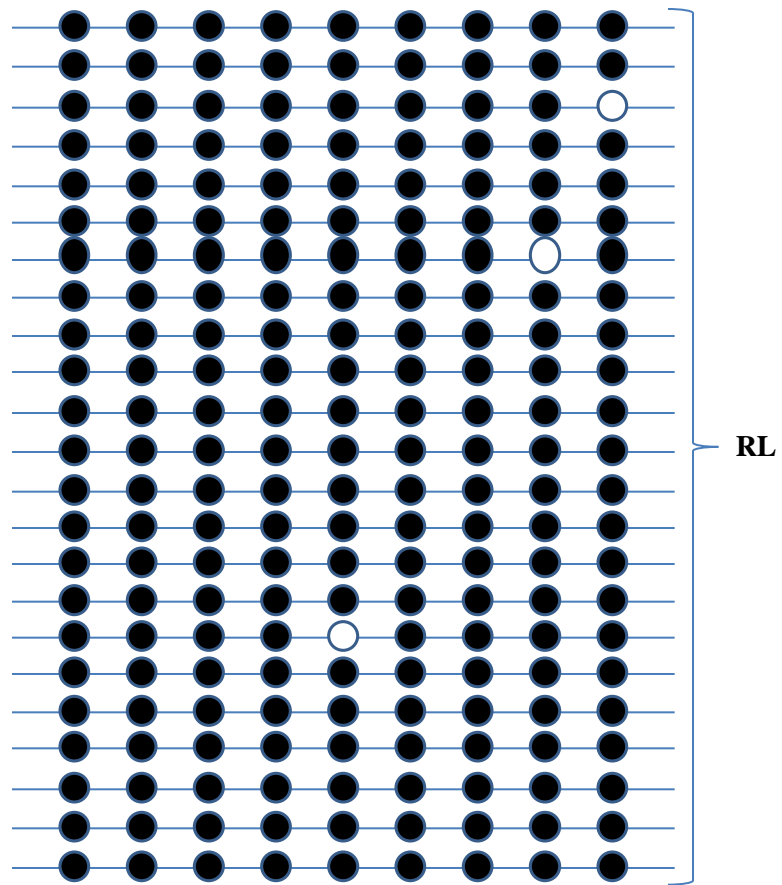

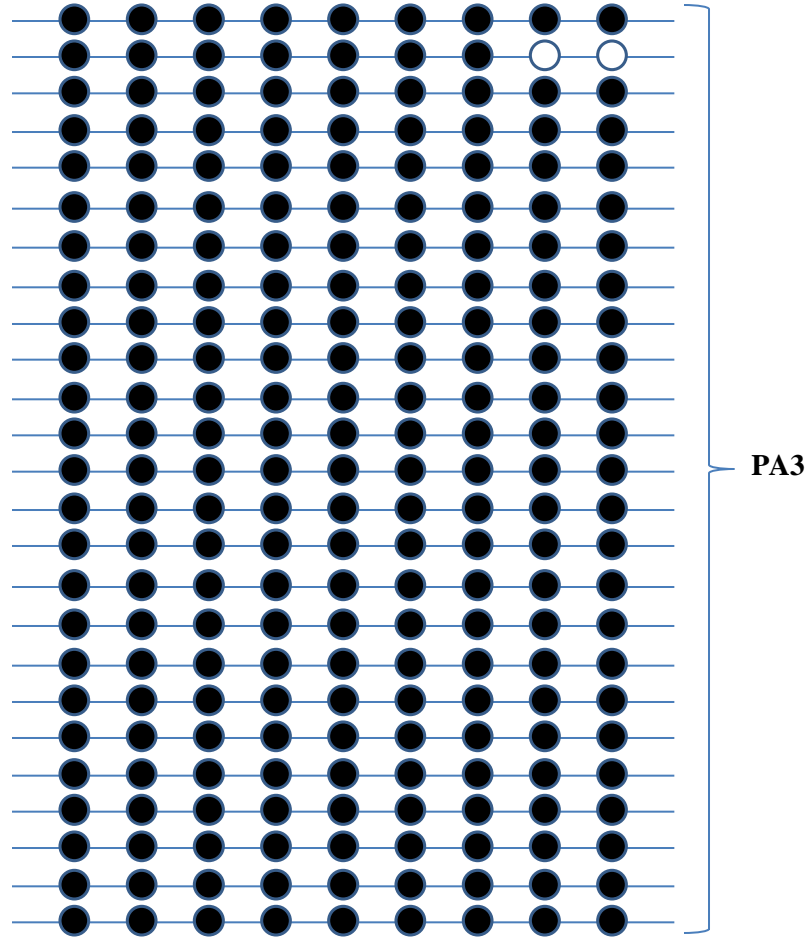

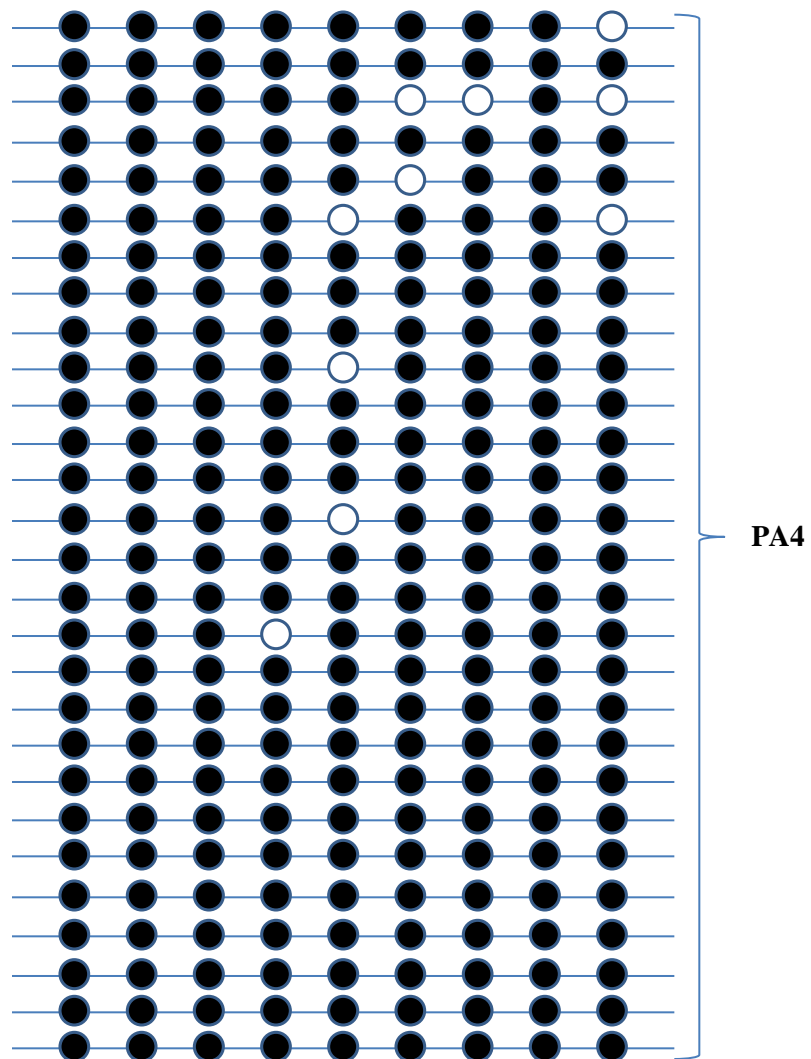

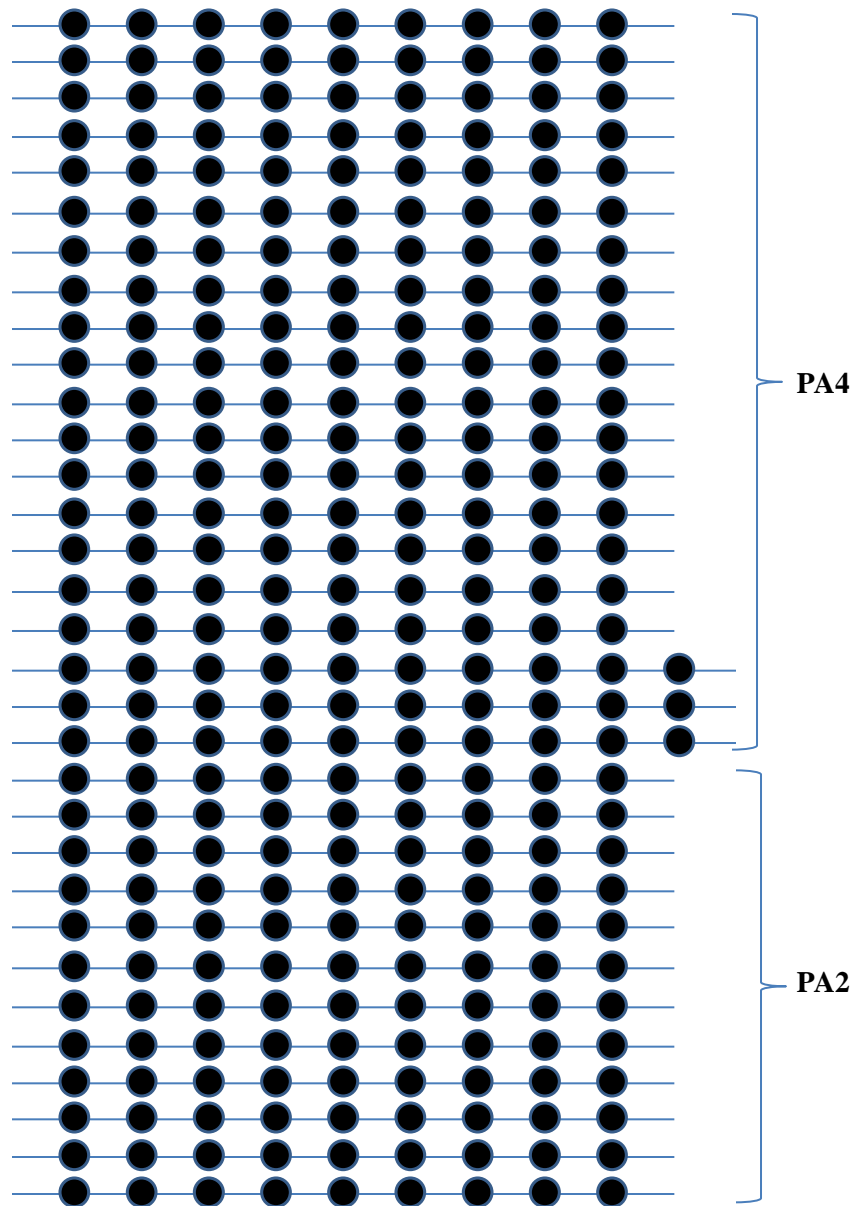

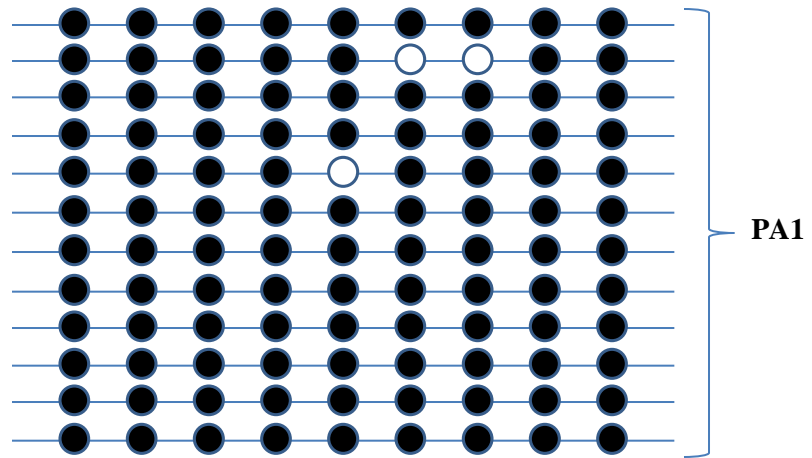

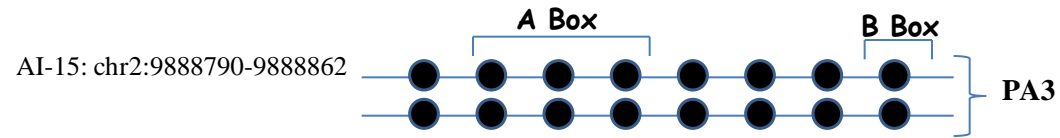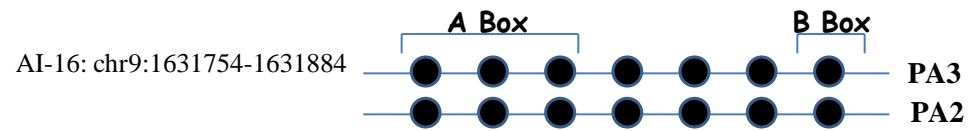

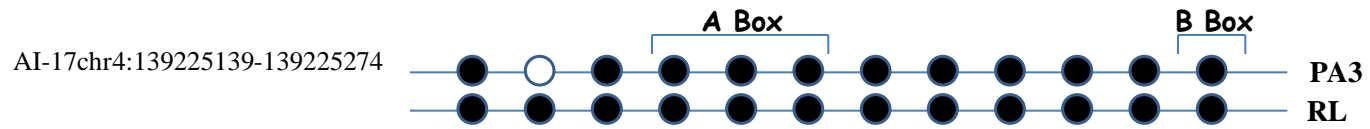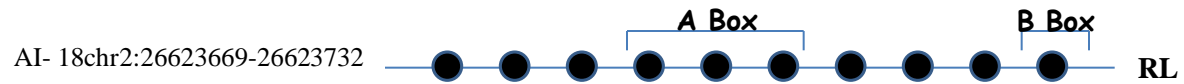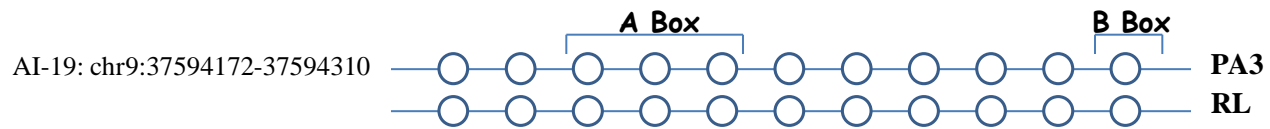

Supplement: Additional file 3 — Figure S1. Methylation pattern of recent Alu insertions. To determine the methylation status, the sequences corresponding to the first half of Alu elements plus its 5' flanking regions [31,32] were aligned to the Alu element sequences generated in this study. NC1: Normal cerebellum and NC2: normal 4th ventricle lining tissue; PA1, PA2, PA3, PA4, and PA5: primary ependymoma tumor; RL: ependymoma tumor relapsed from PA3. [file 1471-2164-12-617-S3.PDF]
