# Supplementary material for: Genetic and epigenetic variations contributed by Alu retrotransposition
Source: BMC Genomics. 2011 Dec 20;12:617. doi: 10.1186/1471-2164-12-617 (PMC3272032; doi:10.1186/1471-2164-12-617)
Supplement: Additional file 4 — Table S3. Alu methylation level. Methylation levels of mapped and 19 non-mapped Alu elements. [file 1471-2164-12-617-S4.DOC]

| **Sample ID** | **NC1** | **NC2** | **PA1** | **PA2** | **PA3** | **PA4** | **PA5**** | **RL** |
| --- | --- | --- | --- | --- | --- | --- | --- | --- |
| Average methylation level mapped Alus* | 95% | 94% | 93% | 94% | 92% | 93% | 91% | 92% |
| Methylation level for the 19 loci verified | 88.8% | 97.6% | 96.8% | 96.2% | 96.5% | 97.9% | - | 98% |

*Average methylation level was determined as the mean of methylation levels of CpG dinucleotides with at least ten sequence reads generated.

** Sample PA5 was used in a previous work in the generation of a methylation map for a subset of Alu elements (Xie et al, 2010), it was not used for verification of new insertions conducted in this study.
