# Supplementary material for: Genetic and epigenetic variations contributed by Alu retrotransposition
Source: BMC Genomics. 2011 Dec 20;12:617. doi: 10.1186/1471-2164-12-617 (PMC3272032; doi:10.1186/1471-2164-12-617)
Supplement: Additional file 5 — Table S4. Validation of identified Alu elements. Primers designed for Alu elements validation. [file 1471-2164-12-617-S5.DOC]

| Sample | Chromosome coordinate | Gene | Primer pairs | | Tm (^0^C) |
| --- | --- | --- | --- | --- | --- |
|  |  |  | Left primer | Right primer |  |
| AI-1 | chr15:61216453-61216621 | RORA | GGGTTGAGTTACATTTATTCAGTAC | CTTTAGTTTTCTTCTTTGTTCAAATCTG | 58 |
| AI-2 | chr5:139595078-139595241 | C5orf32 | GCTCTCACACAGAGAAAC | GTTAACTGCTATCTCTGGGC | 56 |
| AI-3 | chr4:41598260-41598327 | LIMCH1 | CCGATCATTTACATGTAAACGTAC | CATCATTTTCCAGAGACAGC | 52 |
| AI-4 | chr13:23662855-23663027 | - | GACATGGAGAAAGAACTTTGG | CTATGTCCTCAGTTCCTTTAGAG | 51 |
| AI-5 | chr15:28179309-28179438 | OCA2 | CATTTTAAAAAGGAAGATGGGG | GAGAAATCTCATTGACTTCTCC | 52 |
| AI-6 | chr12:32076361-32076491 | - | CATGGGACATGACTTCCT | CTCCATTTTGGATTTCTTTTCAG | 53 |
| AI-7 | chr11:130675880-130675924 | - | GACAAGGACAATGTGGC | CTTCCTACCCCCAGC | 55 |
| AI-8 | chr5:141758572-141758694 | - | CTTTCAAAGTAAAACTAAGCAGG | GGTTACACTTCACAAAGCAAG | 49 |
| AI-9 | chr10:107891481-107891638 | - | GAATCGATTGGTGAGGC | GAGAGCAGTTCAATACCATTATC | 52 |
| AI-10 | chr10:72605338-72605440 | SGPL1 | CTTCCACCTTTGCTTAGG | CAACACTCACCAAGCAC | 56 |
| AI-11 | chr2:48276482-48276601 | - | TGAGAATGGAGAGTCAGG | CTCAAGATTTAAAGGAATGAGCC | 54 |
| AI-12 | chr5:16716576-16716677 | MYO10 | CCGAGCCTTCCATAATTTC | CACAGATTGAATGTCCCCT | 54 |
| AI-13 | chr12:24518543-24518646 | SOX5 | CAGCTAATCTGCACAAAATTTG | GATCTCTTCAACTTCAGTTAAAAAAG | 52 |
| AI-14 | chr6:57403535-57403610 | PRIM2 | CTTAAGAGGTATAAAAATTGTTGGC | GAGCTGGGTTCAACAC | 52 |
| AI-15 | chr2:9888790-9888862 | - | GCTCTTTCAGGGTTGTATG | CAAGTGCTTAGTCACCTG | 52 |
| AI-16 | chr9:1631754-1631884 | - | CTGCCAAAGGATCAAATTCTAG | GGTACAGCAATATAACTCTTTCAG | 51 |
| AI-17 | chr4:139225139-139225274 | - | GTGTTTTTTAGTATTAGCATATGCTTT | GAATGATTTGCTTAACTCTTGTC | 53 |
| AI-18 | chr2:26623669-26623732 | - | CTATAGCTGTTGGATGGTTTTG | CCGTCCTTTGTGAGTAAG | 51 |
| AI-19 | chr9:37594172-37594310 | - | CTGTCACAGAAGCATCC | GTCAAGAAGGAACATTCTGG | 52 |
| AI-20 | chr6:99872263-99872632 | C6orf168 | GCAGGGTTTTGTCTGATAAATC | CCCAGCCCTATTTTTAAATATTCTA | 49 |
| AI-21 | chr2:145175223-145175483 | BC043549 | GAGGGCAATGAACTATCAG | CTTAGTAGTTAAACCTCTGATTCAC | 49 |
